# Myotendinous junction: a microenvironment favorable for short-term adaptations to resistance training following gastrocnemius muscle atrophy

**DOI:** 10.3389/fphys.2025.1493820

**Published:** 2025-05-14

**Authors:** Lara Caetano Rocha-Braga, Jurandyr Pimentel Neto, Isabella Gomes, Adriano Polican Ciena

**Affiliations:** Laboratory of Morphology and Physical Activity (LAMAF), Institute of Biosciences, São Paulo State University, Rio Claro, São Paulo, Brazil

**Keywords:** joint immobilization, skeletal muscle atrophy, telocyte, satellite cell, collagen XXII

## Abstract

The myotendinous junction (MTJ) is an interface region between the skeletal muscle fibers and the tendon, specialized in force transmission, and has a wide capacity to adapt to different stimuli. Disuse muscle atrophy is a deleterious effect of joint immobilization, which is used as a conservative treatment for bone, muscle, and joint injuries and promotes a significant functional decline. Physical exercise is an effective therapeutic modality in combating muscle atrophy, especially resistance training that promotes hypertrophic responses. We aimed to investigate the plasticity of the MTJ in rats subjected to joint immobilization, followed by resistance training in a short period (7 and 14 days). Forty-eight male *Wistar* rats (90 days old) were used and divided into groups (*n* = 8): Control (C), Immobilized (I), Trained (T), and Immobilized Trained (IT). The MTJ samples of gastrocnemius muscle were collected and processed for morphoquantitative analyses using transmission electron microscopy (MTJ and sarcomeres morphometry) and immunofluorescence techniques for collagen XXII, satellite cells and telocytes. We observed that the I group exhibited a reduction in muscle mass, which was associated with a decrease in the length of sarcoplasmic invaginations and evaginations, as well as reductions in belly and proximal sarcomere length. Conversely, the IT groups demonstrated a progressive increase in muscle mass, with significant improvements from 7 days (*p* < 0.01) to 14 days (*p* < 0.0001). The most pronounced adaptations in sarcoplasmic projections were observed in the IT14 group, which exhibited: a significant increase in the length of sarcoplasmic invaginations (*p* < 0.05); a marked increase in sarcoplasmic evaginations (*p* < 0.001); a substantial enlargement of the belly sarcomere (*p* < 0.0001) and proximal sarcomere (*p* < 0.0001); and a notable expansion of the collagen XXII perimeter (*p* < 0.001). We concluded that the joint immobilization resulted in muscle atrophy due to disuse, which led to a decrease in sarcoplasmic projections in the MTJ, a reduction in the perimeter of collagen XXII, and, consequently, fragility of the region. Short-term training demonstrated positive effects on functional improvement, partial recovery of muscle mass, and induction of hypertrophic responses, indicating positive repercussions for the structural recovery of the myotendinous region.

## 1 Introduction

Skeletal muscle tissue has contractile capacity due to its muscle fiber composition (muscle cells/myofibers) and specialized proteins, mainly in its sarcomeres, which work in association with different cells such as adipocytes, fibroblasts, satellite cells (SC), endothelial cells, and neurons (Said et al., 2017; Ghanemi et al., 2019; Bhaskaran et al., 2020; Rosa et al., 2020).

The distal region of the muscle fiber forms an interface between the muscle and tendon tissues, called the myotendinous junction (MTJ), a highly specialized anatomical region. The MTJ is responsible for the transmission of force between the contractile proteins of the terminal myofilaments and the collagen fibrils of the tendon, thus constituting an integrated and specialized mechanical unit (Charvet et al., 2012).

The MTJ features an integrated interface supported by trans-sarcolemmal systems that create a structural connection (Kojima et al., 2008; Charvet et al., 2012; Amemiya et al., 2023). A distinctive characteristic of the MTJ is the exclusive presence of collagen XXII, which is concentrated at the distal end of the muscle fiber where it interacts with the extracellular matrix (ECM). This deposition is facilitated by adjacent nuclei (Jakobsen and Krogsgaard, 2021).

In situations of muscle homeostasis disturbance, such as atrophy, the tissue is accompanied by a metabolic imbalance that leads to the degradation of myofibrillar proteins, in addition to vascular and neural changes, reduction in the number of sarcomeres in series, loss of muscle strength and restriction in the range of movement (Powers et al., 2012; Dutt et al., 2015; Malavaki et al., 2015; Deane et al., 2024), and in the MTJ it can promote a reduction in the contact interface and a predisposition to injuries in the region (Rocha et al., 2021).

A fundamental feature of skeletal muscle performance is its regenerative capacity, regulated by satellite cells (SCs). Under normal conditions, SCs remain in a quiescent state. However, when homeostasis is disrupted—such as during muscle injury or physical stress—these cells are activated. Through specific signaling pathways, they migrate, proliferate, differentiate, and fuse to facilitate muscle repair and regeneration (Said et al., 2017; Ganassi and Zammit, 2022; Jiang et al., 2024).

The interaction of SC with telocytes through extracellular vesicles and direct contact at the MTJ interface was reported with a nerve injury model (Pimentel Neto et al., 2024). In muscle fibers, the telocyte telopodes extend through a fragmented basal lamina and contact the underlying activated SCs in nerve injury models (Manetti et al., 2019). There is substantial evidence suggesting that MTJ remodeling is directly related to SCs and telocytes. During the regenerative process, telocytes are observed next to stem cell niches and provide support for their proliferation and maturation via intercellular communication (Díaz-Flores et al., 2014).

In the skeletal muscle tissue, telocytes have been identified through the endomysium and perimysium (Ravalli et al., 2021), near capillaries and about the vascular system (Zhang, 2016), evolving nerve endings and associated with the peripheral nervous system (Díaz-Flores et al., 2020; Zhang et al., 2023). In addition, the evidence that telocytes may play an essential role in the signal integration for the regulation and regeneration of muscle fibers (Popescu et al., 2011; Marini et al., 2018).

Exercise interventions, including aerobic and resistance training, inhibit muscle atrophy and injury in physiological and pathological conditions. Structured and periodized physical exercise plays a crucial role in the recovery of muscle morphology, metabolism, and function after periods of disuse (Nicastro et al., 2011; Bamman et al., 2018; Howard et al., 2020). Exercise has significant potential in remodeling the morphology of the MTJ. With the increase in transmission force, there is also an expansion in the contact area due to the increased branching of sarcoplasmic projections (Curzi et al., 2012).

However, the short-term effects of resistance exercise on the morphometry and cellular dynamics related to the regeneration of the MTJ region, particularly following muscle atrophy, remain unclear. Including in the gastrocnemius muscle, one of the primary muscles involved in locomotion. Understanding these short-term adaptations is crucial for developing therapeutic strategies aimed at optimizing the interface’s recovery, potentially reducing the heightened risk of injury prevalent during this critical recovery period. That way, the present study aims to demonstrate the adaptations of the MTJ of Wistar rats to short-term resistance training after a model of muscle atrophy due to disuse in the gastrocnemius muscle.

## 2 Materials and methods

### 2.1 Animals

**FIGURE 1:**
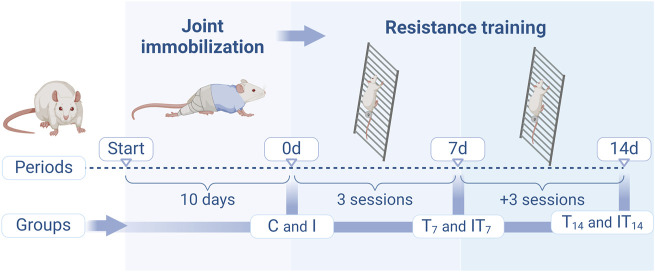
Design of the experimental protocols of the groups in the periods of joint immobilization (10 days), and resistance training (7 and 14 days). Created with Biorender®.

Forty-eight male *Wistar* rats, 60 days of age, were divided into the following groups (Figure 1): Control (C), the animals were not subjected to any protocol (*n* = 8); Immobilization (I): the animals were subjected to the 10-day joint immobilization protocol (*n* = 8); Training (T): the animals were subjected to the resistance training protocol on a vertical ladder, and divided into the subgroups, Training 7 days (T7; *n* = 8) and Training 14 days (T14; *n* = 8); and Immobilization/Training (IT): the animals were subjected to the 10-day joint immobilization protocol and subsequently to the vertical ladder training protocol, divided into the subgroups Immobilization/Training 7 days (IT7; *n* = 8) and Immobilization/Training 14 days (IT14; *n* = 8).

The animals were kept in collective cages, received standard balanced diet (Purina®) and water *ad libitum*, with room temperature controlled at 23°C ± 2°C, with a light/dark photoperiod of 12 h. All procedures applied in this study were previously submitted for consideration and approved by the Ethics Committee on the Use of Animals (CEUA) of the Institute of Biosciences of the São Paulo State University, Rio Claro–SP, Brazil (N° 01/2022).

### 2.2 Joint immobilization protocol

The animals of I, IT7, and IT14 groups were previously anesthetized with Ketamine (90 mg/kg) and Xylazine (10 mg/kg) intraperitoneally and immobilized in the tibiotarsal joint in maximum plantar flexion of the pelvic limb. The device was composed of a stainless-steel mesh cut out with edges covered with adhesive tape, which wrapped the animal’s pelvic limb and kept its right hind paw immobilized. To protect the animal skin the stainless-steel mesh device was filled with cotton. For the fixation of the immobilization device, a cross-band adhesive tape was used to join the cotton fabric wrapping to the steel mesh. The animals remained immobilized for 10 days (Coutinho et al., 2002; Yoshihara et al., 2016; Rocha et al., 2020a). Subsequently, at the time of removal of the immobilization device, the I group was euthanized, and groups IT7 and IT14 continued with the resistance training protocol.

Animals that dislodged the immobilization device and exhibited signs of edema or inflammation were excluded from the experiment. The immobilization protocol restricted movement solely in the immobilized paw, allowing free movement of the other limbs within the cage to ensure adequate circulation and access to food and water. The established model involved immobilization in plantar flexion, leading to progressive shortening of the gastrocnemius muscle throughout the immobilization period.

### 2.3 Resistance training protocol

The T7, T14, IT7, and IT14 groups underwent the vertical ladder training protocol (10 cm high/80° inclination/2 cm between steps). The animals underwent prior adaptation to the resistance training protocol, with 3 sessions with a progression load. The training protocol consisted of 4–9 climbs, with a 2-min interval between each climb. The animals used a load attached to the root of the tail of 50%, 75%, 90%, and 100% of their body mass, respectively, during the first 4 climbs. From the 5th climb onwards, an additional 30 g of weight was added until the animal was exhausted or until the 9th climb (Example: 5th climb = 100% + 30 g; 6th climb = 100% + 60 g) (Krause Neto et al., 2013). The training sessions occurred 3x/week. The T7 and IT7 groups protocol occurred over 7 days, with 3 sessions. Meanwhile, for the T14 and IT14 subgroups, the training sessions occurred over 14 days and consisted of 6 sessions.

### 2.4 Skeletal muscle mass

The samples of the right gastrocnemius muscle were dissected, and the muscle mass data was obtained using a semi-analytical balance (Marte Científica® AD330, Brazil). In order to normalize gastrocnemius muscle mass to total body weight, the following equation was employed: (muscle mass × 100)/body mass. This result is expressed as a measure of relative muscle mass (%).

### 2.5 Histology analysis

The animals were euthanized through an overdose of the anesthetic Ketamine (200 mg/kg) and Xylazine (50 mg/kg) intraperitoneally (*n* = 5 per group). The samples of the belly of the gastrocnemius muscle were collected. Transversal sections (10 µm) were performed (Cryostat—HM 505 E, MICROM®, Ramsey, MN United States) collected on histological slides. Subsequently, the slides were stained with hematoxylin-eosin (HE) to identify cellular components. Some of the collected histological slides (*n* = 6) were processed using the myofibrillar ATPase technique, following the method described by Rocha et al. (2020b). Images were acquired using a light microscope Olympus BX51 with a DP54 lens (Olympus Corporation - Tokyo, Japan).

We quantified the cross-sections area (CSA) of muscle fibers (*n* = 100/group) using the ImageJ™ software. For image acquisition and measurement, we employed a random labeling approach for the histological slides to minimize bias when photographing and quantifying the images.

### 2.6 Transmission electron microscopy

The animals (*n* = 3 per group) were anesthetized with Ketamine (90 mg/kg) and Xylazine (10 mg/kg) intraperitoneally and perfused with a modified Karnovsky fixative solution containing 2.5% glutaraldehyde and 2% paraformaldehyde in 0.1 M sodium phosphate buffer solution and pH 7.3 (Duro et al., 2012). The MTJ samples (3 mm^3^) of the gastrocnemius muscle were dissected and fixed in the same aqueous solution for 3 h at room temperature. Then, the samples were washed with sodium phosphate buffer solution and post-fixed with 1% osmium tetroxide solution for 2 h at 4°C. Semi-thin sections were embedded in resin (Low Viscosity Embedding Media Spurs Kit Electron Microscopy Sciences, Sigma®-Aldrich, Saint Louis, MO, United States). Subsequently, ultra-thin sections (90 nm) were obtained and collected on 200-mesh copper screens (Leica® Ultracut UCT, Göttingen, Germany) contrasted with 4% uranyl acetate solution for 3 min and 0.4% lead citrate aqueous solution for 3 min (Ciena et al., 2012). The screens were examined in a JEOL1010 (Philips® CM 100, Eindhoven, Netherlands) at the Laboratory of Electronic Microscopy Applied to Agricultural Research (NAP/MEPA-São Paulo University ESALQ/USP, Piracicaba, SP, Brazil).

#### 2.6.1 Morphometric analysis

The measurements of sarcomere lengths and MTJ were conducted using transmission electron micrographs. The sarcomere lengths were the distance between the Z-lines pair. For the muscle belly, 200 sarcomeres were measured. Adjacent to the MTJ, measurements focused on two specific sarcomeres: the proximal sarcomere, defined as the last sarcomere near the MTJ with a distinct pair of Z-lines (*n* = 32–64), and the distal sarcomere, defined as the ultimate sarcomere of the myofilament, comprising the basal lamina involved in the sarcoplasmic evagination and the first adjacent Z-line (*n* = 36–65). Measurements of the lengths (*n* = 72–100) and thicknesses (*n* = 90–110) of the sarcoplasmic invaginations and evaginations, as well as the contact interface (*n* = 17–29), were performed at the MTJ. The sarcoplasmic invagination was defined as the region delimited by the tendinous projection, while the sarcoplasmic evagination was defined as the region delimited by the muscular projection. The length measure was determined from the apex of the invagination or evagination to its base. Thickness measurements were taken at three distinct points across the same invagination or evagination to ensure consistent and accurate evaluation. The contact interface was the length of the basal lamina following the contact area between the invaginations and evaginations, measured within a 2 µm line. The measurements were made with the ImageJ software (National Institutes of Health, Bethesda, MD, United States).

### 2.7 Immunofluorescence

The MTJ region of the gastrocnemius muscle collected (*n* = 5 per group), as previously described in topic 2.5, was used. Longitudinal sections (10 µm) were performed (Cryostat—HM 505 E, MICROM®, Ramsey, MN United States) collected on silanized histological slides and outlined peripherally with a hydrophilic pen (PAP PEN ®). The slides were then permeabilized with phosphate-buffered saline (PBS) with 0.1% Triton X-100 (Sigma®-Aldrich, Saint Louis, MO, United States) for 10 min at room temperature. Subsequently, the samples underwent the blocking process in PBS solution with 5% Normal Goat Serum (NGS) for 30 min at room temperature. After 3 washes of PBS for 5 min, the samples were incubated overnight (16 h) in a solution containing primary antibody anti-Pax7 conjugated with Alexa Fluor 488 (1:500, SC81648, Santa Cruz Biotechnology, INC., Dallas, TX, United States), anti-CD34 (1:500, PA585914, Invitrogen®, Oregon, OR, United States), anti-Col22a1 (1:1000, A11008, Invitrogen®, Oregon, OR, United States), and 1% Bovine Serum Albumin (BSA) at 4°C. The slides were then washed 3× for 5 min with PBS and incubated for 1 h with a solution containing secondary antibody anti-Rabbit Alexa Fluor 594 (1:1000, A11012, Invitrogen), anti-Rabbit Alexa Fluor 488 (1:1000, A11008, Invitrogen®, Oregon, OR, United States) and 1% BSA at room temperature. After that, the slides were washed 3× for 10 min with PBS and mounted with ProLong Gold® mounting medium (Molecular Probes™, Eugene, OR, United States) with DAPI for nuclear staining, subsequently sealed, and stored at −20°C. To locate the MTJ we used the phase contrast filter (DIC). The images were obtained through the Olympus® BX61 (Olympus® - Fully Motorized Fluorescence Microscope, Shinjuku, Japan) fluorescence microscope of the Bacterial Genetics Laboratory (LGB), São Paulo State University - UNESP, Rio Claro, SP, Brazil.

#### 2.7.1 Collagen XXII analysis

To measure collagen XXII, the region of interest was identified using the phase contrast (DIC) channel, focusing on the interface where the muscle fiber interacted with the tendon. Images were captured at ×40 magnification and subsequently binarized (conversion of grayscale images into two-pixel intensity levels) based on a threshold to highlight the area of interest, using ImageJ software (National Institutes of Health, Bethesda, MD, United States) (Jones et al., 2016). The area and perimeter of collagen XXII were quantified for each muscle fiber.

### 2.8 Statistical analysis

After obtaining the data, the outliers were identified using the ROUT method (Q = 1%) to ensure the validity of the dataset by removing potential extreme values that could skew the results. Besides that, we used normality tests to assess the data distribution for selecting the most appropriate statistical test. Depending on the outcome of the normality assessment, the statistical test was chosen to ensure robust and accurate analysis (Table 1). The significance level was set as *p* < 0.05 to establish the threshold for statistical significance. All analyses were conducted using GraphPad Prism 8.0.2®, following rigorous statistical principles to guarantee reliable and interpretable results.

**TABLE 1:** Statistical analysis applied.

Variables	Normality test	Statistical test	Pos-test
Skeletal muscle mass (g)	Yes	Anova One-Way	Bonferroni
Skeletal muscle mass (%)	Yes	Anova One-Way	Bonferroni
Cross section area of muscle fibers (mm^2^)	No	Kruskal–Wallis	Dunn’s
Length of sarcoplasmic invaginations and evaginations (µm)	No	Kruskal–Wallis	Dunn’s
Thickness of sarcoplasmic invaginations and evaginations (µm)	No	Kruskal–Wallis	Dunn’s
Interface (µm)	No	Kruskal–Wallis	Dunn’s
Sarcomere length (µm)	No	Kruskal–Wallis	Dunn’s
Collagen XXII area (µm^2^)	No	Kruskal–Wallis	Dunn’s
Collagen XXI perimeter (µm^2^)	No	Kruskal–Wallis	Dunn’s

## 3 Results

### 3.1 Muscle mass

**FIGURE 2:**
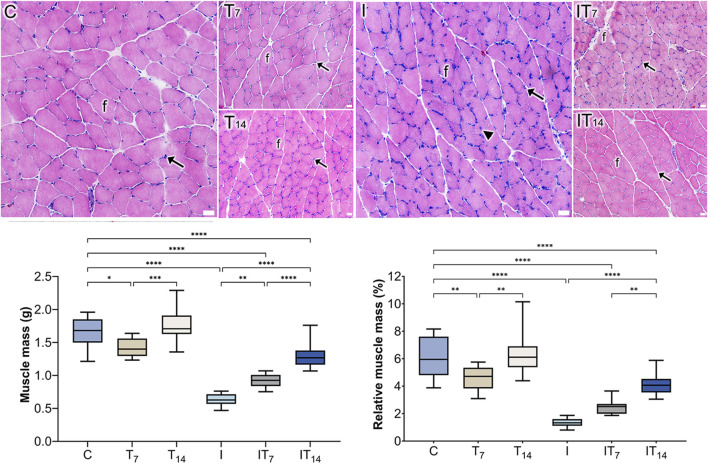
Histological analysis of the groups using the HE technique revealed the presence of muscle fibers (f) and peripheral nuclei (arrows). In the I group, a centrally located nucleus (arrowhead) was observed. Bars: 25 µm. Means and standard deviations of gastrocnemius muscle mass (g) and relative muscle mass (%). *p < 0.05; **p < 0.01; ***p < 0.0005; ****p < 0.0001.

After the analysis, we observed that the T7 group had a lower muscle mass, with 14.4% less compared to the C group (*p* < 0.05), while the T14 group did not show a statistical difference compared to the C group (4.2%; *p* > 0.05). However, a greater muscle mass was observed in the T14 group compared to the T7 group (*p* < 0.0005). In the I group, we observed a lower muscle mass of 62.6% compared to the C group (*p* < 0.0001). Meanwhile, the IT7 group had a muscle mass 48.3% higher compared to the I group (*p* < 0.0001). In the IT14 group, we observed a greater muscle mass of 111.2% compared to the I group (*p* < 0.0001), as well as a higher value compared to the IT7 group (*p* < 0.0001) (Figure 2).

After the analysis of relative muscle mass (%), we observed that the T7 group had less values compared to the C group (*p* < 0.01), while the T14 group did not show a statistical difference compared to the C group (*p* > 0.0001). However, a greater relative muscle mass was observed in the T14 group compared to the T7 group (*p* < 0.01). In the I group; we observed a lower muscle mass compared to the C group (*p* < 0.0001). Meanwhile, the IT7 group had a higher muscle mass but did not show a statistical difference compared to the I group (*p* > 0.05). In the IT14 group, we observed a greater muscle mass compared to the I group (*p* < 0.0001), as well as a higher value compared to the IT7 group (*p* < 0.01) (Figure 2).

### 3.2 Histology analysis

In the C and T7 groups, peripheral nuclei were observed relative to the muscle fibers. In the T14 group, a higher density of nuclei was observed. In the I group, the muscle fibers were smaller, with peripheral nuclei, with a nucleus appeared centralized; however, their distribution was uneven throughout the tissue. In the IT7 group, rounded fibers with peripheral nuclei were observed. In the IT14 group, we identified blood vessels and a bundle of peripheral nerve fibers within the muscle tissue, along with muscle fibers exhibiting peripheral nuclei (Figure 2).

#### 3.2.1 Cross-section area of muscle fibers

In the analysis of the CSA of muscle fibers, the T7 group showed a 24.4% greater CSA compared to the C group (*p* < 0.01). In the T14 group, the CSA was 8.0% lower than in the C group, though this difference was not statistically significant (*p* > 0.05). The I group exhibited a 52.8% smaller CSA compared to the C group (*p* < 0.0001). In the IT7 group, CSA was 128.6% greater than in the I group (*p* < 0.0001). Similarly, the IT14 group showed a 97.6% larger CSA compared to the I group (*p* < 0.0001) (Table 2).

**TABLE 2:** Means ± standard deviations of muscle fibers (mm^2^) cross section area (CSA) of experimental groups with *P*-values.

Groups	Mean ± standard deviations	*P*-value
C	2.002 ± 0.483	—
T7	2.491 ± 0.837	T_7_ ≠ C (*p* < 0.01)
T14	1.840 ± 0.555	—
I	0.943 ± 0.401	I ≠ C (*p* < 0.0001)
IT7	2.156 ± 0.828	IT_7_ ≠ I (*p* < 0.0001)
IT14	1.864 ± 0.609	IT_14_ ≠ I (*p* < 0.0001)

### 3.3 TEM - MTJ morphology

**FIGURE 3:**
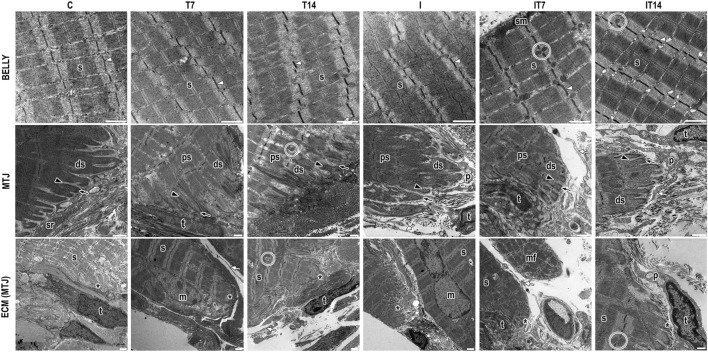
Ultrastructural analysis of the belly muscle and myotendinous junction (MTJ) region by transmission electron microscopy. We observed in the muscle belly the sarcomeres in series (s) delimited by the z-lines (white arrowhead) and the presence of subsarcolemmal mitochondria (sm) and intermyofibrillar mitochondria (circle). In the myotendinous region, we presented the sarcoplasmic projections as invaginations (black arrowhead) and evaginations (black arrow) that compose the MTJ (*); in this region, the proximal (ps) and distal (ds) sarcomeres. In the ECM adjacent to the MTJ, we identified the presence of SR (sr), telocyte (t) with its telopods (white arrow), and pods (p). Besides that, we showed a muscle fiber in a regenerative process in a new muscle fiber in the myotendinous region (mf) and a myonuclei (m) in some experimental groups. Bars: 1 µm.

In the C group, the sarcomeres in series were identified in the muscle belly with well-defined bands and aligned Z-lines. The MTJ presented a conical shape, with its sarcoplasmic projections, invaginations organized in parallel with varying lengths, and the evaginations formed by the distal sarcomeres anchored in the basal lamina. We identified vesicles in the collagen support layer (CSL), the sarcoplasmic reticulum (SR), and telocytes adjacent to the MTJ (Figure 3).

In the T7 group, we observed the sarcomeres in series in the muscle belly with well-defined bands and slight misalignment between the Z-lines. In the MTJ, the sarcoplasmic evaginations had well-defined ends organized in parallel, myonuclei and intermyofibrillar vesicles at the ECM, telocytes, and their projections surrounding the CSL and between muscle fibers (Figure 3).

In the T14 group, sarcomeres in series were observed in the muscle belly with well-defined bands and slight misalignment between the Z-lines and intermyofibrillar mitochondria. In the MTJ region, the sarcoplasmic invaginations presented no defined end but were organized in parallel. The sarcoplasmic evaginations were formed by the poorly defined distal sarcomere but with the presence of the Z-line. The proximal sarcomeres presented a parallel spacing between them with an amount of intermyofibrillar vesicles. Besides that, we observed at the extracellular matrix a telocyte and telopods surround the MTJ in the CSL and a large amount of SR cellular activity (Figure 3).

In the I group, sarcomeres in series are highlighted in the muscle belly without defined bands, as well as misaligned Z-lines. In the myotendinous region, a nucleus is observed. The sarcoplasmic invaginations present ramifications and collagen fibrils inside them (Figure 3).

In the IT7 group, sarcomeres in series were revealed in the muscle belly with well-defined bands and aligned Z-lines. In the region of the muscle belly, the presence of intermyofibrillar mitochondria in the sarcomeric triads, as well as subsarcolemmal mitochondria, is evident. At the MTJ, the sarcoplasmic invaginations did not present good definitions. The telocytes observed in the CSL are adjacent to the SR, in addition to the telopods of other telocytes around the MTJ (Figure 3).

The IT14 group shows the sarcomeres in a series of muscle bellies aligned and with their definitions between bands and lines, as well as their triads. In the myotendinous region, the sarcoplasmic invaginations with an elongated appearance and with a conical shape at their ends. While the sarcoplasmic evaginations presented rounded edges, the distal sarcomeres were longer and without definitions, and in the region, many intermyofibrillar vesicles were also observed. At the CSL there is the presence of oblique collagen fibrils and telocytes with a greater density of pods among the other experimental groups, the telopods that enter a micro tendon in projection to the sarcoplasmic invaginations (Figure 3).

#### 3.3.1 TEM - MTJ morphometric analysis

**FIGURE 4:**
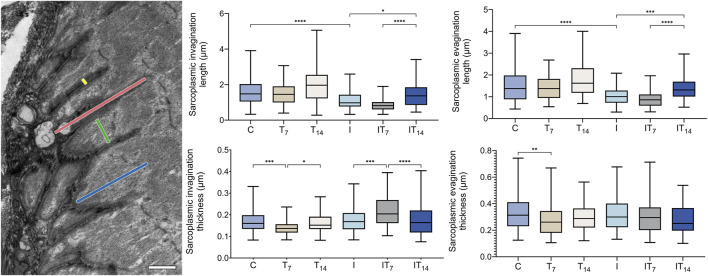
Morphometrics analysis of sarcoplasmic invaginations and evaginations of the experimental groups. Transmission electron micrograph of the MTJ, depicting sarcoplasmic invaginations and evaginations. The length and thickness of the sarcoplasmic invaginations are indicated by red and yellow lines, respectively, while the length and thickness of the sarcoplasmic evaginations are represented by blue and green lines, respectively. Bar: 1 µm. Means and standard deviations of the length of the sarcoplasmic invaginations. Means and standard deviations of the thickness of the sarcoplasmic invaginations. Means and standard deviations of the length of the sarcoplasmic evaginations of the. Means and standard deviations of the thickness of the sarcoplasmic evaginations of the experimental groups. *p < 0.05; **p < 0.01; ***p < 0.001; ****p < 0.0001.

In the morphometric analysis of the lengths of sarcoplasmic invaginations (Figure 4), we observed in the T7 group no significant changes compared to the C group (6.1%; *p* > 0.05), in the T14 group 22.4% greater length value was observed, however, without a significant value compared to C group (*p* > 0.05). The I group presented a lower value of 30.2% compared to the C group (*p* < 0.0001). In the IT7 group, a shorter length of sarcoplasmic invaginations was observed in 23.5% however not statistically significant (*p* > 0.05) compared to the I group. The IT14 group showed a greater length of 42.8% compared to the I group (*p* < 0.05), in addition to a longer length compared to the IT7 group (*p* < 0.0001).

In the morphometric analysis of the lengths of the sarcoplasmic evaginations (Figure 4), we revealed in T7 group no significant changes compared to the C group (9.2%; *p* > 0.05), in the T14 group 15.1% greater length was observed without significance when compared to C group (*p* > 0.05). The I group presented a lower value of 34.9% compared to the C group (*p* < 0.0001). In the IT7 group, a shorter length of the sarcoplasmic evaginations was observed in 14.9%, without statistical significance (*p* > 0.05) compared to the I group. The IT14 group demonstrated a greater length of 34.6% compared to the I group (*p* < 0.01), in addition to a longer length compared to the IT7 group (*p* < 0.0001).

In the morphometric analysis of the thickness of the sarcoplasmic invaginations (Figure 4), we observed lower values of 16.1% in the T7 group compared to the C group (*p* < 0.001), while in the T14 group, there was no difference compared to C group (4.1%; *p* > 0.05), but between T7 and T14 has (*p* < 0.05). The I group demonstrated similar thicknesses to the C group with 5.3% of the difference, but no statistical difference (*p* > 0.05). The IT7 group presented a difference of 23.2% compared to the I group (*p* < 0.001). Meanwhile, in the IT14 group, thickness was observed without statistical difference with I groups (*p* > 0.05). However, the IT7 group presented greater thickness than the IT14 group (*p* < 0.0001).

**FIGURE 5:**
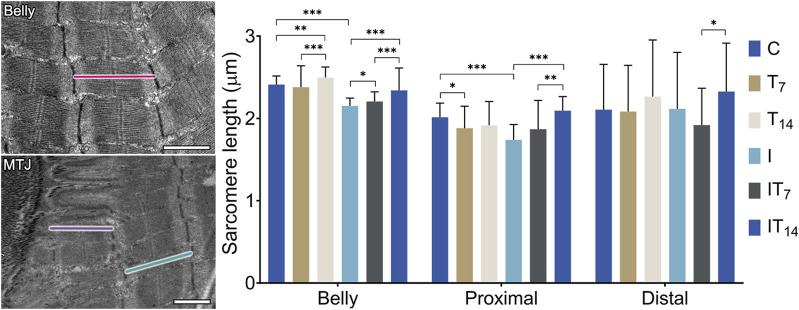
Morphometry of the sarcomere length of the muscle belly and myotendinous region. Bars: 1 µm. Means ± standard deviations of the sarcomere length of the belly (red), proximal sarcomere (purple), and distal sarcomere (green). *p < 0.05; **p < 0.001; ***p < 0.0001.

Through transmission micrographs of the muscle belly and the MTJ region, measurements of the sarcomeric lengths of the experimental groups were performed (Figure 5). In the muscle belly, we observed the T7 group with similar length sarcomeres compared to the C group (1.3%; *p* > 0.05), while the T14 group presented longer sarcomeres by 3.5% (*p* < 0.01), and between T7 and T14 groups there was a difference (*p* < 0.0001). In the I group, we observed shortened belly sarcomeres by 10.7% compared to the C group (*p* < 0.0001). The IT7 group presented a greater length compared to the I group by 2.4% (*p* < 0.05). In the IT14 group, we observed greater lengths compared to the I group at 8.6% (*p* < 0.0001).

In the MTJ region, the penultimate sarcomere determined as the proximal sarcomere was shorter in the T7 group compared to the C group by 6.6% (*p* < 0.05), while group T14 had a similar length to the C group (4.9%; *p* > 0.05). The I group demonstrated shorter sarcomeres in 13.6% compared to the C group (*p* < 0.0001). The IT7 group had greater lengths than group I (7.4%; *p* > 0.05), but without a statistical difference. The IT14 group presented proximal sarcomeres 19.7% longer than the C group (*p* < 0.0001), as well as longer than the IT7 group (*p* < 0.005).

Also, in the MTJ region, the last sarcomere is determined as the distal sarcomere, which presents attenuated variation presented by the standard deviation of the experimental groups. The T7 (1%; *p* > 0.05) and T14 (7.5%; *p* > 0.05) groups presented a value similar to the C group, as did the I group (0.5%; *p* > 0.05). The IT7 group presented shorter distal sarcomeres than the I group (9.4%; *p* > 0.05) without statistical difference. The IT14 group presented larger distal sarcomeres compared to groups I (9.9%; *p* > 0.05) without statistical difference. The IT14 group presented longer sarcomeres than the IT7 group (*p* < 0.05).

### 3.4 MTJ fluorescence analysis

#### 3.4.1 Satellite cell and telocytes

**FIGURE 6:**
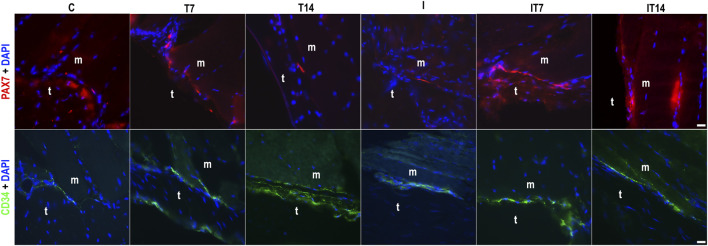
Analysis of the myotendinous region by fluorescence microscopy of the myotendinous junction (MTJ) highlighting muscle (m) and tendon (t) tissue, with the identification of satellite cells (PAX7+DAPI) and telocytes (CD34+DAPI) across the experimental groups. Bars: 20 µm.

The analyses were performed with positive antibodies anti-Pax7 for SCs and anti-CD34 for telocytes (Figure 6). In the C group, we identified the presence of SCs in the perimeter of the MTJ adjacent to the muscle fiber. The telocyte analysis identified their presence in the MTJ. The T7 group demonstrated SCs and telocytes in the perimeter of the MTJ, both in greater numbers than the C group. In the T14 group, the presence of an SC in the MTJ was evident, while the telocytes demonstrated a greater density organized in the perimeter of the MTJ. In the I group, the large presence of nuclei with attenuated sizes in the distal portion of the muscle fibers was noteworthy, and the SCs were present in the perimeter of the MTJ, while the telocytes were in a clustered form in the CSL. In the IT7 group, we observe an amount of SC in the perimeter of the MTJ adjacent to telocytes in the region. In the IT14 group, we found the presence of SC in the perimeter of the MTJ and adjacent to the muscle fiber, like in the analysis of telocytes at the MTJ.

#### 3.4.2 Collagen XXII

**FIGURE 7:**
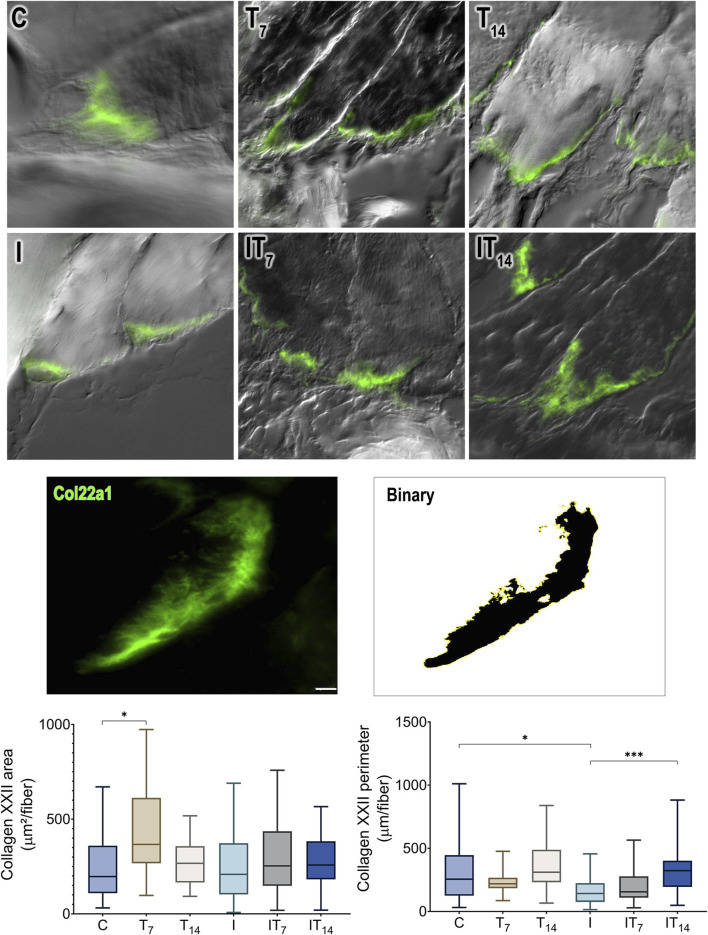
Morphometric analysis of collagen XXII (Col22a1) of the MTJ. Immunofluorescence and binarized image of collagen XXII as an example for morphometric analysis. Bar: 10 µm. Means ± standard deviations of the collagen XXII area of the MTJ (µm^2^/muscle fiber). Means ± standard deviations of the collagen XXII perimeter of the MTJ (µm/muscle fiber). *p < 0.05; ***p < 0.001.

In the collagen XXII area of MTJ (Figure 7), we observed a higher value in the T7 group compared to the C group at 80.0% (*p* < 0.05), while the T14 group showed a value 10% higher compared to the C group, without a statistical difference (*p* > 0.05). The I group showed a collagen XXII area similar to the C group (2%; *p* > 0.05). The IT7 group showed a value 26.9% higher than the I group, without a statistical difference (*p* > 0.05). The IT14 group showed a value 15.7% higher than the I group without a statistical difference (*p* > 0.05).

By measuring the collagen XXII perimeter, we revealed that the T7 group had a 27.3% lower value than the C group without a statistical difference (*p* > 0.05), as did the T14 group (*p* > 0.05). The I group demonstrated a reduced perimeter of 27.7% compared to the C group (*p* < 0.0001). The IT7 group had a larger perimeter compared to the I group by 27.7% but without statistical significance (*p* > 0.05). The IT14 group had a larger perimeter of 96.7% than the I group (*p* < 0.0001).

## 4 Discussion

Through the present results, it was possible to identify the resistance training effects in 7 and 14 days after the induction of muscle atrophy due to disuse caused by joint immobilization. In the MTJ, great cellular activity was observed, with plasticity of sarcoplasmic projections, impact on collagen XXII, and increased CS and telocyte activities.

In the I group, we observed a difference of 62.6% compared to the C group, demonstrating muscle atrophy due to disuse after 10 days of joint immobilization, and muscle fibers atrophy. Muscle inactivity promotes the block of the protein synthesis pathway and the stimulation of autophagy, which negatively impacts the cell volume that makes up the skeletal muscle (Bodine, 2013; Nunes et al., 2022).

The training performed over 7 days resulted in an improvement in muscle mass, with an even more pronounced effect 14 days after removal of immobilization. In the post-immobilization period, muscle tissue becomes particularly susceptible to various stimuli due to homeostatic imbalance also observed with muscle fibers CSA recovery. In this context, training plays a fundamental role in reversing the observed loss of muscle mass (Glass, 2005).

Under normal conditions, myonuclei are uniformly distributed along the periphery of myofibers, beneath the plasma membrane, excluding regions such as the neuromuscular junction and the myotendinous junction—areas where nuclei cluster and are classified into two populations enriched for specific transcripts such as Col22a1 and Ankrd (Roman and Gomes, 2018; Skoglund et al., 2023).

Centrally located myonuclei, indicative of myonuclear mispositioning, are commonly observed in certain muscle disorders, such as muscular dystrophy (Pedrazzani et al., 2021), which is characterized by ongoing cycles of muscle degeneration and regeneration (Roman and Gomes, 2018). Similar, though less severe, central nucleation can also be seen under conditions such as hindlimb unloading (Itai et al., 2004), as observed in the I group.

The presence of central nuclei is widely accepted as a morphological hallmark of muscle regeneration, indicating that the myofiber has undergone a degeneration–regeneration cycle (Hastings et al., 2020). This was evident in the IT7 and IT14 groups, as compared to the I group, with a notable reduction in central nuclei.

The relocation of resident myonuclei to a central position during adaptation to exercise, as part of the muscle repair process (Murach et al., 2020), may explain the findings in the IT7 and IT14 groups, which are also supported by variations in CSA of muscle fibers.

Central myonuclei are considered transcriptionally active during muscle repair in response to severe injury (Buckley et al., 2022; Bagley et al., 2023).

In dystrophic conditions, muscle fibers typically exhibit wide variation in fiber size, central nucleation, basophilic fibers, and necrosis. However, these pathological features can be mitigated by promoting more homogeneous muscle fiber trophism, which leads to reduced variation in fiber size (Pedrazzani et al., 2021)—a pattern we also observed in the IT7 and IT14 groups.

Finally, evidence indicates that IGF-I overexpression can increase the number of central nuclei by 6-fold under immobilization conditions, reflecting enhanced satellite cell fusion with myofibers (Stevens-Lapsley et al., 2010). Although a similar mechanism may be involved in the current protocol, the morphological impact observed was comparatively modest.

In the sarcomeres analysis, contractile units of the muscle fiber, presented varying lengths depending on their location. In the muscle belly, the sarcomeres in series demonstrated greater lengths with resistance training, increasingly in the 7 and 14 days, indicating an adaptation that optimizes contractile performance corroborated by Leite and Rassier (2020).

Joint immobilization, on the other hand, resulted in a reduction in sarcomere length, due to shortening of the gastrocnemius muscle. Okita et al. (2004) also observed this reduction 1 week after immobilization, with no additional changes after 2, 4, 8, and 12 weeks. The shortening of fibers during immobilization can lead to muscle contracture (attenuation of elasticity), where collagen adapts, organizing the endomysium fibrils in circumferential form, and aggravating muscle contracture in advanced stages of immobilization.

After immobilization, training resulted in a progressive increase in the lengths of sarcomeres in the muscle belly after 7 and 14 days. Hinks and Power (2024) reported that the longitudinal morphology of muscles, especially the regulation of sarcomeres in series, adapts more rapidly than the parallel morphology (CSA) during recovery from immobilization, with a slow CSA recovery, requiring greater long-term attention to restore functional capacity.

The sarcomeres of the MTJ showed greater variation in lengths, with the proximal sarcomeres showing a reduction in the T7 group, but no adaptation in T14. The results of Rocha et al. (2020b) partially corroborate this, in an analysis of the plantaris and soleus muscles of rats subjected to 8 weeks of resistance training, it was observed that the proximal sarcomeres have less variation in length than the distal ones, although in the plantar muscle, with a predominance of type II fibers, the proximal sarcomeres present greater length. This possibly reflects differences between short- and long-term training.

In the I group, the sarcomeres showed a significant reduction, with an increase after training (IT7 and IT14). These results contrast with those of 4-week aquatic remobilization studies, in which the proximal sarcomeres showed no changes (Rocha et al., 2021). Resistance and aerobic training activate different metabolic pathways: resistance, of an anaerobic nature (fast and intense), and aerobic, of an oxidative nature (sustainable and prolonged).

The distal sarcomere exhibited wide variation in length in the experimental groups, with a significant difference between IT7 and IT14. With aerobic swimming training, Jacob et al. (2019) identified the MTJ as a region prone to sarcomerogenesis, especially in the distal sarcomeres.

In the sarcoplasmic projections of the I group, we observed a significant reduction only in their lengths. This corroborates Curzi et al. (2012), who reported the interface reduction between MTJ tissues and a response relative to the type of muscle fiber and the duration of the condition that promotes atrophy. The atrophy in the MTJ is complex due to its constant maintenance for the transmission of contractile force by protein synthesis while facing a process of generalized catabolism in the muscle fibers. This balance between the need to preserve contractile function and the wear induced by atrophy creates a challenging scenario for maintaining the structural integrity of the MTJ.

In the IT7 group, compared to the I group, there was only a change in the thickness of the sarcoplasmic invaginations. According to Egan and Sharples (2023), acute adaptations to exercise occur up to 48 h after practice, with transcriptional changes and subsequent morphological adaptation after 7 days. Immobilization establishes a state of predisposition to external stimuli, becoming an important moment for remobilization methodologies. Thus, the adaptation observed in the MTJ of the IT7 group reaffirms the adaptive potential of the MTJ and training as a therapeutic agent for the interface.

The IT14 group, compared to the I and IT7 groups, already presents greater lengths of sarcoplasmic invaginations and evaginations. Kojima et al. (2008) observed an increase in tension in the MTJ, and an increase in the angle formed by the interface and the muscle fiber, which can cause transient adaptation and an immediate response to increased tension during exercise.

In the morphometric analysis of the MTJ, a larger contact interface was observed in the T7 group compared to the C group, while the other groups presented more pronounced variations, suggesting the non-uniformity of the sarcoplasmic projections, responsible for forming a continuous anchoring base. Jakobsen et al. (2023) analyzed the MTJ of type I and II muscle fibers, revealing that type I fibers have an interface area 22% larger than type II fibers. The gastrocnemius muscle has type II fibers predominance, and in our morphometric analyses of the MTJ, the fiber types were not differentiated.

The SC observed through immunofluorescence analysis (Pax7+) exhibited different arrangements according to the experimental protocols. During the quiescent state, SCs are located under the basal lamina and receive signals that maintain the quiescence of adjacent skeletal muscle fibers (Wong et al., 2021). However, in situations of muscle disorders, such as disuse atrophy, the composition of the SC niche undergoes significant changes, allowing the emergence of new contacts that mediate proliferation, self-renewal, and differentiation (Kann et al., 2021).

Pimentel Neto et al. (2024) recently described the first relationship between telocytes and SCs at the MTJ in a nerve injury model, where paracrine activity was observed between these cells, in addition to interactions mediated by vesicles, a mechanism similar to endocrine signaling, but acting over short distances by passive diffusion. This paracrine communication occurs in the ECM of the MTJ and extends to the sarcolemma. The telocytes identification and SC in the same region reveal a close relationship in the microenvironment formation favorable to rapid adaptations, considered a potential approach for understanding tissue regeneration (Rosa et al., 2021). In this study, the presence of telocytes and SC in the MTJ was observed, evidencing their potential in analyses of fast tissue regeneration.

Telocytes were observed in the MTJ in different arrangements, with a higher prevalence around the myotendinous interface. Pimentel Neto et al. (2020) identified telocytes in the MTJ in niches close to capillaries, pericytes, and collagen fibers in the CSL. With resistance training, telocytes perform their paracrine activity, promoting the remodeling of sarcoplasmic projections. The influence of telocytes, associated with short-term training after joint immobilization, contributes to tissue regeneration and repair. Telocytes play an essential role in remodeling by acting as communicating agents, using complex mechanisms of electrical, chemical, and epigenetic communication, including the exchange of exosomes, to coordinate activities between different types of cells and tissues (Edelstein et al., 2016).

Collagen XXII (Col22a1+), observed in the T7 group, showed a greater area occupied at the end of the muscle fiber, while its perimeter showed a minor variation, similar to the C group. This result indicates a higher concentration of collagen XXII in a smaller region. Charvet et al. (2013) described the behavior of the interface in zebrafish with depletion of Col22a1, which resulted in a phenotype similar to muscular dystrophy, with impaired functional capacity, significant reduction in the amplitude of muscle contractions, and detachment of fibers induced by contractions. In addition, they demonstrated that the absence of collagen XXII caused a significant reduction in sarcoplasmic projections. In the I group, the area of collagen XXII showed no changes compared to the C group, but the perimeter was smaller, reflecting a less homogeneous arrangement. Miyamoto-Mikami et al. (2020) suggest that high expression of Col22a1 mRNA in the MTJ may influence the risk of muscle injury in athletes due to two alleles associated with collagen XXII, although they were unable to determine the exact location of the injury.

The IT7 group did not show significant changes compared to the I group, while the IT14 group exhibited a larger perimeter than the I group, suggesting a more dispersed distribution across the muscle fiber contact interface with the basal lamina. Our results corroborate those of Jakobsen et al. (2017), who reported the presence of collagen XXII in the human MTJ after 4 weeks of training protocol, but not in muscle or tendon, reinforcing the functional specialization of this region. Furthermore, the authors highlight the increase in the collagen XIV and Tenascin-C in the endomysium near the MTJ, suggesting a remodeling of the matrix, possibly for protection against strain injuries. Our results indicate that collagen XXII is essential for maintaining the structural integrity of the MTJ, and its modification may result in functional and morphological changes that compromise muscle performance and mechanical stability during contraction.

The sample size may have contributed to a higher standard deviation, potentially reducing the statistical power of the study, and thus representing a limitation of this research. Additionally, the quantification of centralized nuclei in the immobilized groups was not performed due to the lack of adequate standardization, as the limited number observed did not allow for sufficient image uniformity required for reliable comparative analysis. Nevertheless, the presence of such nuclei may indicate that the myofibers have undergone a degeneration–regeneration cycle, and the signs of this process should also be considered when interpreting the observed histological features.

We conclude that at the MTJ, resistance training promoted cellular activity growth and variation in the plasticity of sarcoplasmic projections, highlighting the variability of the MTJ sarcomeres. SCs and telocytes are present at the MTJ, revealing a close relationship with a microenvironment formation favorable to short-term adaptations. Collagen XXII presents as a key factor to different conformations, indicating the adaptability in making the interface more compact.

## Data Availability

The original contributions presented in the study are included in the article, further inquiries can be directed to the corresponding author.
